# Exploring factors that affect nurse staffing: a descriptive qualitative study from nurse managers’ perspective

**DOI:** 10.1186/s12912-024-01766-7

**Published:** 2024-01-31

**Authors:** Xiaoyan Yu, Miqi Li, Meichen Du, Ying Wang, Yu Liu, Hui Wang

**Affiliations:** Department of Nursing, Tongji Hospital, Tongji Medical College, Huazhong University of Science and Technology, No. 1095 Jiefang Avenue, Wuhan, 430030 China

**Keywords:** Nurse staffing, Nurse manager, Semi-structured interviews, Thematic analysis

## Abstract

**Background:**

The appropriate nurse staffing reflects the situation of nursing management of human resources. Nurse managers have a pivotal role in determining a competent and sufficient number of nurses. It is important to understand the factors influencing nurse staffing to promote appropriate staffing levels. The study aimed to explore the factors affecting nurse staffing from the perspective of nursing managers.

**Methods:**

Purposive sampling was adopted to recruit 14 nurse managers from secondary and tertiary hospitals located in the central region of China, and semi-structured interviews via telephone were conducted from April to May 2022. Interview transcripts were analyzed and collated using thematic analysis.

**Results:**

This research identified four themes and ten subthemes influencing nurse staffing. Extracted themes include: government level (inadequacy of mandatory policies, budgetary constraints), hospital level (hospital characteristics, the control of nurse labor costs, inadequate support on nursing), patient level (patient characteristics, increasing care needs), and nurse level (nurse shortage, skill-mix, individual high-level needs).

**Conclusion:**

The findings indicate that it is crucial for decision-makers or policymakers to legislate for safe nurse staffing and establish effective supervision and funding incentives. Tailored interventions are also needed to improve the organizational context, address the nurse workforce and balance the structure of nurse staff.

**Supplementary Information:**

The online version contains supplementary material available at 10.1186/s12912-024-01766-7.

## Background

Many countries have achieved significant development in healthcare personnel resources in recent years [1, 2]. The nurse staff is considered an integral part of the healthcare workforce and makes up approximately 60% worldwide [3]. While the world has credibly acknowledged nurse staff as vital in helping prevent adverse outcomes, promoting patients’ health and improving their satisfaction with healthcare services, one of the main challenges faced is the global shortage of nursing workforce [3–5]. By the end of 2020, the number of registered nurses (RNs) in China was 4.70 million, or 3.34 RNs per 1,000 population [6], which is much lower than the average of 9.4 RNs per 1,000 population in the member countries of the Organization for Economic Co-operation and Development (OECD) [7]. Moreover, it reported that the bed-to-nurse ratio in general units was 1:0.53, and over 70% of nurses had a junior college degree or above [6]. With the development of the economy, increased coverage of medical insurance networks, the three-child policy, and an ageing population with growing healthcare needs, the healthcare system in China faces enormous challenges, such as the shortage of nursing workforce and the imbalanced skill structure of nurse staff [6, 8].

Nurse staffing is referred to the number of nurses, professional qualification of nurses, nurse-to-patient ratio or skill-mix [5, 8]. Optimal nurse staffing is the concern of most nurse leaders worldwide and is essential for patient safety and quality of care [9]. In 2016, the World Health Organization (WHO) raised a vision for health care: accessible, acceptable, quality, and cost-effective health care with the staffing of nurses according to patients’ expectations and needs [10]. Studies have shown that appropriate nurse staffing helps to ensure better patient outcomes and improve the quality of healthcare, including shorter length of hospital stay, lower levels of in-hospital mortality and hospital-acquired infections, as well as fewer omissions of nursing care [11–13]. To support nurses empowered to create staffing plans specific to each unit, some parts of the world, such as California, the United States, Victoria, and Queensland state, Australia, have passed the law for the minimum nurse-to-patient ratio, which has been proven to have benefits for patients and healthcare system [9, 14]. In China, there are two standards for nurse staffing in clinical settings, including the bed-to-nurse and nurse-to-patient ratios, which are requirements for the total allocation of nurses to a ward and for given shifts in a ward, respectively [15]. In 2012, the Ministry of Health of China recommended that the patient-nurse ratio in general wards should be no more than eight [15]. The requirement for the bed-to-nurse ratio in general units should be not less than 0.55 nurses per bed [8]. Though there are three shifts for nurses in most Chinese hospitals, nurse staffing standards have not been established for different shifts [16]. Furthermore, these two indices are the only requirements on the number of nurses, without considering nurses’ working experience, professional titles, or educational levels.

Nurse staffing is essential to the ever-evolving healthcare system, and high baseline staffing levels are needed to enhance patient health. Previous studies reported several factors, such as the increasingly aging workforce, changing workplace climate, and high nursing workloads, continue to drive insufficient nurse staffing in most hospitals across the world [17, 18]. In China, the healthcare system is different from most Western countries in terms of government healthcare expenditure, public health insurance, and a tiered healthcare delivery system. The government healthcare expenditure in China is underfunded, accounting for 6.5% of gross domestic product [19]. Hospitals may not enroll the healthcare workforce, particularly the nurse staff, when facing financial challenges. The nurse manager is pivotal in determining the unit’s nurse number and skill mix of nurses [20]. Those managers should be able to logically suggest the requisite number of nurses who can give enough care to patients. While studies consistently emphasize the importance of sufficient nurse staffing levels [21, 22], the staffing of nurses in hospital settings is more complex than solely acting on evidentiary support. Therefore, the objective of this study was to explore the factors influencing nurse staffing from nurse managers’ perspective. This result could help identify the obstacles in the allocation of nursing resources and highlight the need for an appropriate nurse staffing plan involving all relevant stakeholders.

## Method

### Design

A descriptive qualitative with semi-structured interviews via telephone was used to explore the factors influencing nurse staffing from nurse managers’ perspective. The consolidated criteria for reporting qualitative research (COREQ) checklist was followed to ensure the quality of research [23] (see Table S1).

### Setting and sample

This qualitative descriptive study was conducted in 5 secondary and 9 tertiary hospitals in the central region of China. Purposive sampling was used to achieve a maximum variation in nurse managers until data reached theoretical saturation (i.e., no new themes were identified) [24]. The eligible criteria were nurse managers with at least five years of experience in that position at a public general hospital. Participants were excluded if they were not willing to participate in the research. Before the data collection, one researcher (MQL) introduced the research purposes and procedures for eligible participants, who were contacted by E-mail or telephone. They were invited to participate in the interview if they showed interest in this research.

### Data collection

A semi-structured interview guide based on research purposes was developed to direct conversations toward the research topic through an extensive review of relevant literature, following the process recommended by Kallio et al. [25]. A meeting with the research team was held to revise the interview guide by removing ambiguous questions. Next, an ethics specialist was invited to assess the appropriate wording. The final interview questions in the interview guide are presented in Table S2. The interview guide was piloted with two participants prior to the formal interview. There was no need to reformulate the questions, so the pilot data were included in the analysis. Telephone interviews were conducted by two authors (XYY and MQL), and the time for interviews was set according to the participants’ convenience and preferences. All enrolled participants were asked to complete a written informed consent form and provide their relevant demographic data before being interviewed. The interviews lasted from 15 to 40 min. The interview data were collected from April to May 2022.

### Data analysis

We used thematic analysis to scrutinize data [26]. The data collection and data analysis were simultaneously conducted. Two independent researchers repeatedly read transcripts and notes to understand participants’ exact meanings (XYY and MQL). Key lines and condensed meaning units were highlighted in the text, which were coded to generate initial codes. Similar codes were clustered to create subcategories and categories, which were grouped into themes. The data analysis was ongoing throughout data collection. The first author determined the initial coding, and the others read a sample of coded interviews to check the coding. All authors discussed the assigned codes several times until they reached a consensus. Following data analysis, the emerged themes and interview excerpts were translated into English by the researcher (bilingual in English and Chinese), and then back-translated into Chinese by a translator to ensure their exact meaning were consistent with the original transcripts [27].

### Rigor

The research was conducted and reported by the following four criteria to ensure rigor: credibility, transferability, confirmability, and dependability [24]. For the credibility, the whole interviews were held using a semi-structured interview question, and field notes were taken throughout the interviews. To ensure the confirmability, data were analyzed by two independent researchers [28]. We also conducted member checks with two participants separately to review and comment on interpretive notes via online face-to-face meetings [28]. To establish dependability, verbal data were recorded and interpreted. In the meantime, relevant quotations were also attached to elaborate on each theme and subtheme. Lastly, transferability was supported by providing contextual information such as age, hospital type, and a detailed description of the interview data collection process [28].

### Ethical consideration

The hospital granted permission to conduct the study (number: TJ-IRB20220454). All enrolled participants were informed about the study procedure. Written informed consent was obtained from participants before the interview. They were informed that participation was entirely voluntary and had the right to withdraw from the research at any time without negative consequences. Besides, all data were confidentially maintained. Only the researchers and research team had access to the data in a password-protected computer.

## Results

A total of 14 participants participated in the research, and none refused or withdrew. All participants were women with a mean age of 48.71 years (43 to 58 years) and an average work experience of 29.5 years (24 to 36 years). Half of the participants held a master’s degree. Nice participants came from tertiary and five from secondary hospitals. The participants’ demographic characteristics are shown in Table 1. Four themes emerged from the interviews: government level, hospital level, patient level, and nurse level. Participant’s quotations were used as exemplars to illustrate the critical issues experienced by participants and to support each theme and subtheme.

**Table 1:** Characteristics of the fourteen nurse managers

No.	Hospital type	Gender	Age (years)	Education level	Professional title	Working experience (years)
NM1	Secondary hospital	Female	48	Bachelor	Co-chief superintendent nurse superintendent nurse	29
NM2	Secondary hospital	Female	49	Bachelor	Chief superintendent nurse	30
NM3	Secondary hospital	Female	51	Bachelor	Co-chief superintendent nurse	33
NM4	Secondary hospital	Female	54	Bachelor	Chief superintendent nurse	36
NM5	Secondary hospital	Female	49	Bachelor	Co-chief superintendent nurse	30
NM6	Tertiary hospital	Female	51	Bachelor	Chief superintendent nurse	33
NM7	Tertiary hospital	Female	43	Bachelor	Co-chief superintendent nurse	24
NM8	Tertiary hospital	Female	50	Master	Co-chief superintendent nurse	33
NM9	Tertiary hospital	Female	43	Master	Co-chief superintendent nurse	24
NM10	Tertiary hospital	Female	43	Master	Co-chief superintendent nurse	26
NM11	Tertiary hospital	Female	50	Master	Chief superintendent nurse	31
NM12	Tertiary hospital	Female	58	Master	Chief superintendent nurse	31
NM13	Tertiary hospital	Female	46	Master	Co-chief superintendent nurse	27
NM14	Tertiary hospital	Female	47	Master	Chief superintendent nurse	26

### Government level

#### Inadequacy of mandatory policies

Nurse staffing policy requires the hospital to determine a minimum average staffing level in a ward. However, the policy’s detailed operation, including the punishment detail and special operation, may affect the enforceability.

Seven interviewees in the study mentioned that some mandatory measures should be adopted to ensure sufficient staffing levels. One participant said:“We want to comply with the nurse staffing policy, …it seems that there are no mandatory or punishment measures, for example, if it could not achieve the nurse staffing levels…”(NM11). Another participant stated: “If the nurse staffing levels are related to…the evaluation of hospital quality indicators, the hospital managers would pay some attention to nurse workforce …”.(NM 13).

Besides, the nurse staffing standards have some limitations in the complex clinical context. Accordingly, the following narratives were recorded:

For example, some departments such as the cardiology department, which belongs to internal medicine, have set its bed-to-nurse ratio… However, there is no detail in the policy document …. (NM 10)

The bed-to-nurse ratio was 1:0.4, which was published in 1978. Until high-quality care was proposed in 2010, it recommended the ratio was 1:0.5. (NM 4)

#### Budgetary constraints

Financial support from the government was the primary incentive mechanism for hospitals to increase nurse staffing levels. However, budgetary constraints at a governmental level may affect the nurse staffing. The following narrative highlighted these findings:

Our hospital (secondary hospital) has a relatively low level of nurse staffing, and it is necessary for the hospital to control labor costs for further development…without enough economic support, and now it adopt contract-based employment, these nurses will feel the same as a part-time job…they would rather find a good job outside the hospital, and may not feel so burnout….(NM 5)

If nurses’ salary is subsidized by the government, we are willing to… increase nursing human resources.(NM 9)

### Hospital level

#### Hospital characteristics

Many factors are necessary to be considered in the rational allocation of nursing human resources, such as the hospital size, hospital types, and hospital service capacity. Accordingly, the following narratives were recorded:

We thought the most important factors were the hospital size and its workload, when we set nurse staffing standards. (NM 7)

It is also necessary to consider the hospital type; if the hospital provides service for more elderly people, women or children, the bed-to-nurse ratio may be different. (NM 9)

#### The control of nurse labor costs

In the market-oriented healthcare system, hospitals tend to consider human resource costs, especially the nurse labor costs, which account for the majority of hospital expenditure. The following narrative highlighted these findings:

Heads of department are unwilling to recruit nurses because of its labor costs. (NM 3)

The hospital boards will take it (labor cost) into consideration when in developing stage, … if the total number of patients does not reach a certain level, the hospital boards will control the labor costs and not enroll enough nurses at once. (NM 8)

#### Inadequate support on nursing

Nurse staff is considered an integral part of the healthcare system. However, hospital leaders’ perspectives and their support may affect nurse staffing. The following narratives were recorded:

First of all, hospital leaders’ perceptions on nursing is crucial, some still hold on the view that the nursing care is less important so that priority will be given to…control nurse workforce.(NM 3)

For example, in the anti-COVID stage, the bed-to-nurse ratio reached 1:0.4, because of the deployment of nurse personnel from other medical institutions. However, after that, with the deployment of nurse personnel decreasing, all hospitals began to restore normal order and needed more nurses to deliver care service; however…the hospital leaders thought that they could ensure the hospital’s normal operation, it was not necessary to recruit more nurses. (NM 4)

### Patient level

#### Patient characteristics

The basic characteristics of patients, including total number, age, education level, and economic conditions …, affect nursing workload, so there are requirements for nursing human resources. The following narrative highlighted these findings:

There are many elderly people hospitalized in our hospital. Some of them have low education level, with more healthcare demands, so our nurses spend more time caring for them…. (NM 5)

However, due to the increasing patients and their demand for healthcare, we have to enroll more nurses, and increase labor costs… as a result. (NM 7)

#### Increasing care needs

With the change in health concepts, more patients tend to pursue high-quality nursing services. Moreover, the specific nursing service is also required according to the patient’s disease condition. Accordingly, the following narratives were recorded:

Nursing personnel should be allocated …according to patients’ disease risk and condition, as well as bed-to-nurse ratio.(NM 12)

If there are more serious patients in the department, it means that more nurses are needed, such as advanced practical nurses.(NM 5)

Patients hope to get better quality of care in the hospital…, so they have the feeling of security. Thus…the quality of nursing care has to be improved.(NM 6)

### Nurse level

#### Nurse shortage

The limited nurse number may restrict the reasonable allocation of nurse staff, which is also the main dilemma of the current distribution of nurse human resources.

First of all, the total number of nurses is relatively inadequate, so no matter how we allocate nurses, it could not meet the clinical care needs. (NM 8)

However, the annual nurse recruitment plan is not adopted. In fact, it is very difficult to recruit nurses, so that the units have low baseline staffing. (NM 10)

#### Skill-mix

Nurses’ age, work experience, professional levels and professional title are essential embodiments of nurse structure, which can promote the rational allocation of nursing human resources.

For nurses’ competency level, not just about Level 1, level 2, … we have to consider, such as specialist nurses….(NM 10)

Nurse’ age, working experience, education level, professional title, etc., should be considered in the overall staffing. (NM 11)

#### Individual high-level needs

Nurse individual needs and choices were essential in directing their professional development. The following narrative highlighted these findings:

Some nurses with three-year college degrees are enrolled in our hospital. However, once they complete their undergraduate education, they intend to resign…. (NM 3)

In recent two years, nurses with higher education level are more likely to leave because…they can get better opportunities. (NM 4)

## Discussion

This study offers insights into the multiple factors influencing nurse staffing from nurse managers’ perspectives. Analysis of our qualitative data from 14 nurse managers showed that four factors influenced the complex and dynamic organization of nurse staffing: government level, hospital level, patient level, and nurse level.

The results of this study showed that nurse staffing was affected by mandatory policies and budgetary constraints at a governmental level. Nurse staffing policy could secure a sufficient number of nurses in hospital, which is the vane of nursing human resource allocation. Though the Ministry of Health of China has developed standards, planning or guidance on nurse staffing [6, 15, 29], there is no safe staffing legislation similar to that in Queensland and California where the ratio of no more than five or four (in Queensland) patients per registered nurse is required on medical and surgical wards for the day shift [30]. Moreover, a lack of supervision measures would weaken the policy’s mandate to allocate nurses [31]. On the other hand, budgetary constraints at a governmental level are one of the main factors hampering nurse staffing and the long-term development of the nurse team. In the future, while safe nurse staffing should be legislated, it is also necessary to establish supervisory mechanisms to ensure the effectiveness of policy implementation and to promote the sound development of the nurse workforce. Furthermore, there is a need to establish incentive mechanisms for medical institutions to improve the baseline staffing levels.

The results of this study showed that hospital level, which constituted of hospital characteristics, the control of nurse labor costs, and inadequate support on nursing, was considered an essential factor influencing nurse staffing. Providing nurse staffing levels that match patient healthcare needs is vital to deliver cost-effective health services [13]. However, the nurse staff is the largest staff group, accounting for a large proportion of the hospital’ variable costs, which was sometimes regarded simply as a costly labor input [22, 32]. Furthermore, nursing care may not contribute direct benefits to medical institutions; those leaders may control costs by reducing the number of nurses, decreasing nurses’ income, etc. Therefore, nurse staffing decisions need to address the baseline staff establishment to roster and better respond to fluctuating nurse care demand among patients [13]. Previous studies have reported that the benefits of better nurse staffing extend to nurses as well; those nurses in better-staffed hospitals report less job dissatisfaction, burnout, or intention to leave their jobs [16, 33]. Griffiths et al. found that a higher baseline staffing plan, which was planned to meet 90% of demand, was more resilient in the face of variation and may also be highly cost-effective, due to much of the increased additional staff costs being offset by savings from reduced length of stay in hospital [22].

Nurses constitute the backbone of the health care system, and sufficient nurse personnel is essential to human resource allocation. This study found that nurse shortage, skill-mix, and individual high-level needs were the factors affecting nurse staffing. The nurse shortage is considered a critical global problem, and this concern is further exacerbated by the trend of nurses leaving their positions. Nurse turnover rates vary across countries, with 15.1% in Australia [34], 17.8% in the United States [35], and 23% in Israel [36]. Liu et al. reported that the turnover rate of nurses was 0.64%~12.7% across 22 secondary and 26 tertiary hospitals in Jiangsu Province of China [37]. In such circumstances, nurses are struggling to meet the demands of patients and organizations. A recent study found one third of the Chinese nurses were overworked [8], which may affect the quality of nursing care and cause nurses to increase job dissatisfaction, exhaustion and intention to leave [8, 16, 38]. In addition, due to the rapidly changing healthcare context, it poses a challenge for nurses to update their knowledge and skill, and adapt to the newest medical technology to deliver comprehensive health-care needs for patients [39, 40]. Nurses with different work experience and educational levels may be equipped with distinctive abilities to provide healthcare, which should be considered in nurse staffing [22]. Thus, further consideration should be paid to achieving the optimization of nurse staffing.

### Implications for nursing management

Exploring the factors influencing nurse staffing could present evidence for decision-makers or policymakers to address nurse shortages and promote appropriate nurse staffing to ensure high-quality patient care. An effort should be made to provide supportive measures by reinforcing policy, investing in the nurse workforce, improving the organizational context, and offering nurses’ professional development needs, which, in turn, would increase baseline nurse staff, improve nurses’ work attitudes, and their intention to stay in the medical institution.

### Limitations

Our study has several limitations. First, although we purposively sampled participants to ensure diversity of opinions and experiences, our study conducted a semi-structured interview via telephone, and some information, such as non-verbal data, may be missing during the interview. Second, the interview data were translated from Chinese to English, it is still a risk to misinterpret and mislay some of the code meaning while translating data.

## Conclusions

This study uncovered multiple factors on governmental, hospital, patient and nurse level that may affect nurse staffing. Results illustrate the complexity of the implementation process for nurse staffing, highlighting the need for a well-thought-out nurse staffing plan with the involvement of all relevant stakeholders. Nurse staffing levels across all sectors and settings, and for all shift patterns, should be legislated for safe nurse staffing, and its supervision and funding incentive mechanism should also be established. Tailored interventions focused on improving the organizational context, addressing the nurse workforce and balancing the structure of nurse staff, are needed to improve quality of care and nurse and patient outcomes.

### Electronic supplementary material

Below is the link to the electronic supplementary material.

**Supplementary Material 1: Table S1.** COREQ (Consolidated criteria for reporting qualitative research) Checklist. **Table S2.** Semi-structured interview guide

## Data Availability

The data being used and analyzed during the current study are available from the corresponding authors upon reasonable request.
